# Repeated Low-Level Red-Light Therapy for Controlling Onset and Progression of Myopia-a Review

**DOI:** 10.7150/ijms.85746

**Published:** 2023-09-04

**Authors:** Qin Zhu, Xuejun Cao, Yuan Zhang, Yuan Zhou, Jieying Zhang, Xiaofan Zhang, Yingting Zhu, Liping Xue

**Affiliations:** 1 Department of Pediatric Ophthalmology, Affiliated Hospital of Yunnan University, Kunming 650021, China.; 2 Department of Ophthalmology, the First Affiliated Hospital of Kunming Medical University, Kunming 650031, China.; 3 BioTissue (Tissue Tech, Inc.), Ocular Surface Center, and Ocular Surface Research & Education Foundation, Miami, FL, 33126 USA.

## Abstract

Repeated low-level red-light (RLRL), characterized by increased energy supply and cellular metabolism, thus enhancing metabolic repair processes, has gained persistent worldwide attention in recent years as a new novel scientific approach for therapeutic application in myopia. This therapeutic revolution led by RLRL therapy is due to significant advances in bioenergetics and photobiology, for instance, enormous progresses in photobiomodulation regulated by cytochrome *c* oxidase, the primary photoreceptor of the light in the red to near infrared regions of the electromagnetic spectrum, as the primary mechanism of action in RLRL therapy. This oxidase is also a key mitochondrial enzyme for cellular bioenergetics, especially for the nerve cells in the retina and brain. In addition, dopamine (DA)-enhanced release of nitric oxide may also be involved in controlling myopia by activation of nitric oxide synthase, enhancing cGMP signaling. Recent evidence has also suggested that RLRL may inhibit myopia progression by inhibiting spherical equivalent refraction (SER) progression and axial elongation without adverse effects.

In this review, we provide scientific evidence for RLRL therapy as a unique paradigm to control myopia and support the theory that targeting neuronal energy metabolism may constitute a major target for the neurotherapeutics of myopia, with emphasis on its molecular, cellular, and nervous tissue levels, and the potential benefits of RLRL therapy for myopia.

## Introduction

Myopia is caused by the parallel light penetrating via the eye refractive system and focusing the front retina as the eye is relaxed, which causes blurred images 1. Myopia includes axial myopia due to the axial overgrowth and refractive myopia because of excessive curvature in the cornea and lens 2. As the worldwide accepted, myopia is defined as spherical equivalent (SE) ≤ - 0.5 diopters (D), high myopia is defined as SE ≤ - 5.0 D (or - 6.0 D) or axial length (AL) greater than 26 mm 3-5.

Myopia has increasingly become a major public health problem, causing visual damage worldwide 6. In Asia, for example, myopia is pandemic in China, due to constant increased prevalence in recent decades. In China, approximately 80% school age children suffer myopia when they complete schooling, of whom about 10-20% endure high myopia 7, which may cause serious myopic maculopathy, glaucomatous optic neuropathy and irreversible blindness 8. A meta‑analysis indicates that the prevalence of myopia and high myopia in 2010 was 23% and 3% respectively in the world 8. Unfortunately, the prevalence of myopia may increase to ~50% by 2050, thus 4.8 billion people worldwide may suffer from this disease 8. Therefore, to prevent onset, to slow down progression of myopia have become the important strategies for myopia control in this world.

Myopia is linked closely with many complications, for instance, cataracts, macular degeneration and vision loss 9-12. The causes related to myopia progression include but not limit to high educational pressure 13, little time outdoors 14 and more near-work activity 15 (for detailed reviews, see 1, 16). Children born after 2010 are surrounded with a world through TV, smart phones, computers, which are now classified as causative factors 17, 18.

Previously, various interventions, including atropine, orthokeratology, spectacle and soft contact lenses, defocus incorporated multiple segment (DIMS) lenses and their combinations have been applied to slow down myopia progression with limited success 19-23. For instance, Lam et al have demonstrated clear vision along with constant myopic defocus may reduce myopia progression by inhibiting axial elongation and myopia progression in myopic children 24. Unfortunately, the efficiency for controlling myopia is between 30% to 60% with certain side effects using those methods 25. Other measures to retard progression of myopia with similar efficacy include decrease of near work time 26, increase of time spent outdoors 27, 28 and improvement of scleral hypoxia 29. Recently, more attention has been devoted to myopia prevention and control by red-light exposure, due to its high effectiveness for retardation of myopia with no side effects (reviewed in 30).

## Light and Red-Light

**Light** is defined as a form of electromagnetic radiation manifested by particles and wave properties. The waves of electromagnetic radiation possess unidirectional vectors, specified by wavelengths (λ, successive peak distance), frequencies (oscillations per second), and amplitudes (difference of trough and peak). The energy particles within electromagnetic radiation include photons, which travel at 3×10^8^ m per second. Thus, mixture of various waves would elicit photons moving at different amplitudes and frequencies that are scattered and absorbed, reflected by various objects, for instance, biological materials.

By contrast, red-light is monochromatic, allowing high specificity as to molecular biomodulation. Red-light can be generated by lasers or light-emitting diodes (LEDs), appliable to photobiomodulation (PBM) in eyes. Lasers may generate coherent light energy efficiently to allow penetration to various tissues. The beam width of the lasers can be strengthened by coupling with fiber optic to permit energy distribution to larger areas. LEDs may generate efficient light without coherence in wavelengths of 4-10 nm. However, LEDs generate negligible heat, without any thermal injuries 31. LEDs may also be coupled to the arrays with various functions, to deliver energy effectively to large areas, for instance, to the brain. Therefore, LEDs have been used in many human trials, and the use of LEDs has been approved by FDA 32. Far red (FR) and near infrared (NIR) light generated by LEDs or a laser may promote cerebral blood flow (CBF) 33, 34, stimulate brain energy metabolism 35, 36 and increase the antioxidant capacity 37, mediate cell growth 38 and improve reparative ability in cells 32. NIR and FR may also protect optical nerves, enhance their functional recoveries 39, 40.

## Association of Light Wavelengths with Myopia

More recently, effects of different light wavelengths rather than those of different intensities on myopia onset and progression have drawn increasing attraction. Light waves are electromagnetic with different frequencies, including infrared rays, ultraviolet rays, radio waves, visible light (reviewed in 30). Low frequency of electromagnetic fields (LF-EMFs) with a long wavelength may reduce expression of type I collagen in human fibroblasts and inhibit scleral remodeling, which may inhibit myopia onset and progression 41. Interestingly, differences have been noted in the wavelength of light by different people and therefore visible light wavelengths are not consistent for retardation of myopia for different people 42.

Interestingly, chickens under blue-light may possess hyperopia, while those under red-light may possess myopia 43. An experimental chick model has shown that blue light may retard myopia progression while red-light may enhance myopia progression 44. Violet light at 360-400 nm may inhibit axial elongation in chicks and humans, by decreasing early growth response 1 (EGR1) gene expression known to inhibit myopia 45. Violet light neuropsin (OPN5) in retina may also play a critical role to reduce myopia progression and mediate choroidal thickness in a mouse model 46. Red-light may promote a hyperopic shift while blue light may not influence emmetropization in a mouse model 47. Short wavelength light at 400 nm may delay eye growth, induce hyperopic shift, and prohibit myopia induced by lens 48. In guinea pigs, blue light promotes hyperopic effects and inhibit axial growth while green light promotes myopia progression 49. Positive lens promotes light to focus on the anterior part of retina, enhancing defocus-induced hyperopia while negative lens results in DIM Guinea pigs (reviewed in 30). It is reported that blue light may inhibit axial myopia by negative lens and delay axial growth while red-light and positive lens do not induce hyperopia in a guinea pig model 50. However, there is an inconsistency. For instance, researchers did not find that red-light promotes development of myopia but stimulates hyperopia instead 51. Red-light may inhibit while blinking blue light may promote myopia progression in a tree shrew model 52. Those results indicate that difference of wavelengths from different lights may cause different refractive and axial changes, which may be present independently of light intensity. The results from various species have added complexity in the association between light exposure and myopia 53-59.

## Association of Light Color and Luminance with Myopia

Human color vision requires three types of color vision, short, middle and long wavelength sensitive cones, corresponding to their color perception channels. Precise information on color, light, dark and saturation is essential for two or more contrasts of cone and color vision channels 60. Those signals may be affected by different pathways, such as brightness and 2 color vision alignment pathways 61-63.

Time-related changes from color contrast are defined as hyperopic defocus while time-related changes from luminance contrast are regarded as myopic defocus in absence of color changes 30. The effect of the modulated ambient light on emmetropization have been studied extensively 64-75. Nevertheless, due to species and experimental variabilities, the results reported are quite different. For instance, exposure to 0.5 and 5 Hz modulated illumination chronically has been reported to promote myopia progression in a guinea pig model 72. Steady or flickering red-light may promote hyperopia, flickering blue light may promote myopia while steady blue light may not have any effects 52. Gawne et al have also demonstrated that longitudinal chromatic aberration (LCA) is a critical visual cue of postnatal refractive development, and that short-wavelength temporal flickers are important for assessing and signaling defocus 76. The visual system may use reactivity of longer-wavelength cones to blue light to adjust focus. As a result, blue light may promote emmetropization, and the amount of flicker detected by SWS channel may be more than that by long wavelength sensitive (LWS) channel 52. Spatial contrast may also be an important factor for promoting myopia. In fact, eye may use a single retinal image for comparison of contrast from two different cone types to define state of hyperopia and myopia, without contrasting images in 2 different planes 61, 64. In contrast of a single retinal image, eye may obtain information from comparing changes in color and luminance contrast along with changes from defocus of eye 77. Those results suggest that LCA may reduce cone contrast from L- and M-cones, resembling when eye is in hyperopic or myopia defocus status.

In addition, an association between light luminance and myopia is present. An example is form-deprived myopia (FDM), referring to that induced myopia may be achieved by deprivation of form vision from eyes in a status of susceptibility, for example, lid-suture 78. Low color temperature artificial lighting may retard form-deprived myopia progression in a juvenile monkey model, suggesting that low correlated color temperature (2700 K - 3000K) may delay ocular axial growth, when compared to high color temperature artificial lighting (4000 - 5000 K) 79. One study has suggested that recovery from myopia in a chick model relies on the correlated color temperature of the light spectrum 80. Another example is defocus-induced myopia (DIM) or lens-induced myopia (LIM), referring to myopia induced by concave or negative lenses, for instance, in chickens with lenses in front of eyes 81. Light illuminance from outdoor is usually 10 to 1000 times higher than that from indoor 82, 83. A DIM model has shown that chicks under high intensity light (15,000 lx) retarded development of myopia and slowed progression of myopia than those under low intensity light (500 lx) 84. Similarly, an FDM model has demonstrated that chicks under high intensity light may delay myopia development 85-87. Association of high light exposure and myopia control was later verified in FDM model using monkeys and mice 88, 89. The association among near work, outdoor activity and myopia in school children was explored by Rose et al., the results of which have indicated that outdoor activity may decrease myopia even under high near-vision activity 27. Zadnik et al have shown that outdoor activities may protect children against myopia 90, suggesting that intense light in outdoor environment may play a critical role in regulating onset and progression of myopia. Several studies have shown that the rate of myopia onset and progression are reduced in children with significant more time outdoors 91-93. Unfortunately, the mechanism remains unclear.

## Red-Light Targets and Possible Mechanism

Red light may promote biological processes at a low dose but inhibit biological process at a high dose, in a U-shape response. Hormetic curves are better to predict biological responses within pharmacological threshold than linear curves 94. It is very important since stimulatory responses from low-level red-light are mild, ~ 30% to 60% above controls. The hormetic dose-response is well known in various low-level red-light applications because photo--stimulatory or photo-inhibitory effects are shown in low (0.001 J/cm^2^) or high (0.10 J/cm^2^) energy densities respectively 95. Positive responses may be obtained in that dose range for needed therapeutic effects 32, 96, 97. Examples of low-level red-light effects and applications are listed for our understanding of usefulness of low-level red-light for potential medical applications. For example, wound healing may emerge in the rat skin under a low energy ruby laser 98 and under Gallium laser with 685 or 830 nm light 99. Spinal cord injuries may be ameliorated by 810-nm light in a rat model 100. Low-level red-light was beneficial for treating gingival incisions 101, oral mucositis 102, ulcers of skin 103, and wound healing 104, nerve repair 105-107, carpal tunnel syndrome 108, 109 and soft tissue injuries 110. Inflammation could appear in light-stressed retina 111-113. Microglial inhibitor naloxone may inhibit photoreceptor degeneration within retina 114, 115. Pretreatment with LEDs can prevent neurons from apoptosis induced by cyanide due to inhibition of reactive oxygen synthesis and promotion of energy metabolism 116. 670 nm red light has potential for medical applications reported by many researchers. For example, 670 nm light could protect neuronal cells under treatment of cyanide 32, protect photoreceptors in rat and promote wound healing in primate retina 117, increase of mitochondrial metabolism, decrease of retinal inflammation, and reduction of oxidative cell stress may be achieved probably by changes of respiratory chain complex I, II and IV to affect cytochrome *c* oxidase (CCO), resulting in better energy metabolism within mitochondria 118. As reported, 670 nm red light may be used as an effective neuroprotectant against other light caused damage 119 and certain toxins 120. Treatment with 670 nm red light decreases retinal inflammation by increasing mitochondria membrane potential 121, improves retinal healing 31, such as reducing raised intracellular pressure in rat retina 122, retards aging retinal functions 123. 670 nm LED may regulate inflammation and immunity in the retina of a mouse with macular degeneration 124, likely through promoting CCO expression, along with reduced inflammation 125. Respiration in aged retinal mitochondria may be enhanced by 670 nm light, showing better mitochondrial functions and inflammation reduction through activation of CCO. In fact, aged retina may possess a progressive oxidation increase by 670 nm light in 5 minutes 126. 670 nm light can improve oxygen-mediated degeneration within retina in mouse, showing decreased oxidative stress marker expression and reduction in hyperoxia 127. 670 nm light treatment may significantly retard lipid peroxidation, complement propagation in retina degeneration 128. Low levels of 670 nm light may prevent retinopathy by oxygen induction and lung damage by excessive oxygen 129 and modulate expression of genes involved with inflammation, oxidative metabolism and apoptosis 130. Evidence has indicated that CCO is the primary photo receptor 131, promoting oxidative metabolism 132 and increase ATP production 133, and is probably linked to increased CCO and reduced acrolein expression 134 through the reparative and/or the protective mechanism. In fact, abundant research results supports its potential benefits in retinal diseases 97, stroke 135, 136, neurodegeneration 137, neuromuscular disorders 138, hair regrowth 139, memory 140 and mood disorders 141.

Although many photoreceptors have been discovered, the mechanism of low-level red-light remains unclear since many molecules have photoreceptor capabilities. For instance, reactions from a molecular target induced by a wavelength can be complex. For example, NADH-dehydrogenase and flavoprotein have various photoreceptors from the violet to blue or from red to near-infrared spectral areas 133. In addition, both terminal oxidase and superoxide dismutase have absorption peaks at high wavelengths of 670-680 nm, overlapping in the absorption spectrum of different photoreceptors 133, 142. Those lights may enhance mitochondrial membrane potential, stimulate ATP exchange and production, promote synthesis of RNA, proteins, and usage of oxygen 143. The effects from low-level red-light on mitochondria are wavelength-specific, and molecules absorbing low-level red-light in living cells are likely respiratory components 144. For instance, RNA synthesis from HeLa cells may be enhanced by some wavelengths. The other example is cytochrome oxidase, especially cytochrome *c* oxidase, a primary light photoreceptor in the red and the near-infrared electromagnetic spectrum 145, 146, also a critical oxidase in cell bioenergetics in nerve cells from retina and brain 147. Cytochrome oxidase exerts pump function by redox-coupled protons to stimulate transmembrane electrochemical gradient, synthesizing energy-generating ATP 148. Cytochrome oxidase participates in free radical metabolism, glutamatergic regulation and apoptosis 149, 150. Cytochrome oxidase is also involved in intraneuronal metabolic activity and neuronal functions 150. In fact, cytochrome oxidase can be the only primary photoreceptor by sequential irradiation 151, 152. Interestingly, absorption spectra from cytochrome oxidase is strongly linked to biological responses induced by low level red light 151.

Among the possible hypotheses for relationship of myopia and light intensity, the most possible hypothesis is that the light promotes synthesis and release of dopamine (DA) by retina 153. DA is a critical neurotransmitter in retina, regulating multiple functions, such as refractive development, β receptor activation, visual signal transduction and myopia development, probably by activation of its receptor 154 (also reviewed in 30). In fact, DA receptors are G-protein-coupled receptors present in retina, including D1, D2, D4, and D5 sub-receptors 155. Among the sub-receptors, D2 receptors are more critical in mediation of myopia progression than D1 receptors in chicks (reviewed in 30. Activation of D2 receptors may result in myopia, while activation of D1 receptors may cause hyperopia 155. Light may promote DA release in a linear relationship with 4 log units of intensity 153. DA release from retina may promote choroidal thickening with ocular growth retardation, probably by release of nitric oxide (NO) from retina and choroid to slow down myopia development 156-158. Red, blue and UV light may also promote DA release from retina, with wavelength dependence 159. For instance, blue and UV light may promote less deprivation myopia than red light (reviewed in 30). Circadian rhythms could be another mechanism for light exposure to control myopia, overlapping with that of eye growth, in which DA is a critical mediator for the rhythms 160, 161. Melatonin may also be critical in the association of the light and circadian functions 162. AL and choroidal thickness of eyes may be opposite for the changes with circadian rhythms, which may be interfered by DIM modes, suggesting that optical defocus may play a critical role in regulation of AL and choroidal thickness 163-165. In all, light intensity is negatively linked to onset and development of myopia.

DA may enhance synthesis and release of NO (nitric oxide) to mediate eye growth via activation of NOS (nitric oxide synthase). NO may decrease form deprived myopia dose dependently while NOS inhibitors may block myopia inhibition regulated by atropine 166, 167. Activation of NOS may promote cGMP production to inhibit myopia in guinea pigs 168. PBM may be caused by intracellular NO release 169, 170. NO may inhibit oxidative agents, promote oxygen transport, and hemoglobin production. If combined with oxygen, NO may promote release of oxygen via NO competition 171. Hypoxia and reoxygenation injury of cardiomyocytes may be relieved by 670 nm light, depending on NOS dependent or independent NO 172. PBM may promote NO production via reduction of nitrite to NO mediated by CCO, a critical cytochrome oxidase, and myoglobin (Mb)/Hb 173. CCO is a terminal complex for eukaryotic oxidative phosphorylation within mitochondria, which couples reduction of electron carriers in metabolism to reduction of molecular oxygen into water and proton translocation from internal mitochondrial matrix to inter-membrane 174. A study has indicated that under 628 nm light for 3 days, the expression of the respiratory and antioxidant genes was promoted, as the expression of apoptotic genes is inhibited in human fibroblasts through CCO activation 175. Photoreceptors of mammalian hemoglobin may be activated by certain light, manifesting that light absorption may be a differential sue to the redox state, allowing its quantification of oxygenation in clinical uses. CCO may be activated by light in FR to NIR range, causing cellular responses 32, 133. Catalase, cryptochromes, cytochrome b, cytochrome c, guanylate cyclase, nitric oxide synthase and superoxide dismutase also possess photoreceptors 31.

Red light may induce oxidation in cytochrome c via activating CCO to increase use of oxygen, to elevate mitochondrial membrane sensitivity and pore permeability 133, 142. The effects are mediated by the increase of electron flow in mitochondrial electron transportation chain. Red light may promote production of free radicals, via singlet oxygen due to photodynamic activation and generate superoxide ion through electron auto-oxidation. Red light can generate transient heat from chromophore due to electric or light oscillations 133, which can alter various molecules from the targets. RLRL may also promote energy transfer. Secondary effects from low-level red-light may appear as results of primary effects, for instance, biochemical and biological reactions which affects cellular homeostasis 176-178.

With treatment of monochromatic light, the symmetric myopic sign-dependency is manifested, resulting in the spherical defocus on the ocular acuity. The observed results suggest that the human visual system may integrate the chromatic differences in refraction to discern the signs of defocus 179. Red light can activate signaling transduction from mitochondria to nucleus via activation of photoreceptors and translocation of signaling molecules to the nucleus, to alter gene expression. That is, red light may enhance beneficial NAD/NADH ratio changes in mitochondria to promote nitric oxide release via activation of cytochrome oxidase, and thus regulating ATP level. ATP may then activate ATP P2 receptors to cause inward calcium currents through calcium release from intracellular storages of calcium 180. As a result of such ATP changes, which then mediate level of cAMP, inducing activation of several kinases to mediate gene expression, which can reduce inflammation and improve wound healing.

PBM may also activate TGFβ/Smad signaling in wound healing to mediate myopia progression 181, 182. 670 nm light may promote production of collagen I and vascular endothelial growth factor (VEGF) in wound healing 183. Decrease of collagen I alpha1 (COL1A1) and increase of α-SMA may be linked to sclera hypoxia 29. Therefore, we deduce that FR and NIR treatment may promote choroid thickening and improve blood flow through release of NO acting as a vasodilator. NO may also have anti-hypoxia effect, inducing amelioration of scleral hypoxia. Activation of the TGFβ/Smad pathway may promote scleral remodeling via overproduction of COL1A1. Along with NO, trans-differentiation of sclera fibroblasts may be reversed, without any specific mechanism so far.

## PBM and PBMT

**PBM** or low-level light therapy, via photon irradiation, has decades of clinical applications to soft tissue injuries due to promoting wound healing. Low-level red-light can penetrate tissues such as the brain, heart and spinal cord 184, 185, accelerate healing from ischemic heart injury 184, inhibit degeneration in injured optic nerve and retina 184, 186, and promote recovery in stroke 185, 187, 188. These effects by low-level red-light are clearly associated with the absorption spectrum of cytochrome oxidase 31, by overexpression of cytoprotective factors 32, 116, 185, 186, 189-192, accumulation of antioxidants 120. For example, gene profile analysis shows that low-level red-light promotes noncoding RNAs (ncRNA) to mediate certain gene expression 130. During PBM, cytochrome oxidase, a primary photoreceptor of low-level red-light, plays a critical role in recoveries of injured eye and brain tissues because the enzyme in mitochondria is critical for oxidation. Low-level red-light has significant neuroprotective effects in regeneration of damaged retina via promoting mitochondrial function, improving blood flow to the damaged neurons, promoting expression and release of survival factors from cells 193.

**PBMT** (photobiomodulation therapy) has increasingly become popular in human medical therapies recently. Recent reports have suggested that laser therapy can be used for treatment of inflammation and promotion of wound healing 194. Best of all, energy from PBMT is low without any side effect concern for tissue destruction due to overheating, however, good enough for medical purposes. For instance, PBMT may be applied as photodynamic therapy for skin diseases 195 and as thermal therapy of neuronal diseases 196.

## Red-Light Therapy for Myopia Prevailing

Myopia pandemic affects a large number of population in the world 197-203 (reviewed in 204). Higher Myopia may cause serious consequences such as cataract, choroidal degeneration, glaucoma, retinal tears and detachment 205-209. The RLRL clinical trials are mainly conducted in China due to their recent invention of the new RLRL therapy machine. Xiong et al have shown that RLRL therapy may induce sustained choroidal thickening over the entire course of treatment 210. Liu et al have shown that the choroid's thickening is not sufficient to explain the reasons for AL shortening, which may be linked to changes from the posterior segment 211. Higher aberrations mean optical problems of eyes that alter quality of retinal image, causing visual progressive deterioration. Therefore, various myopia control methods have been developed such as use of orthokeratology, soft contact lenses (dual-focus or multi-focus) and near addition spectacle lenses, which have enormous effects for the world population 212. Yam et al have also shown that 0.05%, 0.025%, and 0.01% atropine may delay myopia progression by concentration-dependent response, and of 3 concentrations used, 0.05% atropine was the best to controlling AL elongation and myopia progression in a 1-year trial 22. Yam et al have later reported that the efficacy of 0.05% atropine is twice as good as that with 0.01% atropine in retarding myopia progression 213. We recently have reported that 1% and 0.05% atropine respectively may delay moderate myopia progression in Chinese school children by a mega clinical investigation 214, 215 and long-term wear of orthokeratology lenses also retard myopia progression 216. However, as our generation is aging, the prevalence of myopia is increasing continually 217 due to their limited effectiveness and obvious side effects. Therefore, we need to find more effective ways to control the development and progression of myopia.

Recently, several reports have indicated that RLRL may inhibit myopia progression by inhibiting SER progression and AL elongation 20, 218-220. Hung et al have suggested that narrow-band with long-wavelength light may inhibit axial elongation typically produced by form deprivation and hyperopic defocus, due to eliciting direction signals related to myopic defocus 212. However, not all the reports are comprehensive and many undiscovered characteristics remain to be explored, for instance, whether RLRL treatment may prevent myopia occurrence from preclinical status, whether such treatment is better than other conventional treatments for myopia, such as atropine, DIMS, multifocal contact lenses and outdoor exercises 91, 213, 221-226. Wang et al have indicated that approximately a quarter school children have had inhibition of AL elongation by RLRL therapy, and the mean AL change is -0.142 mm per year 227. Recently, there are new efficacy reports for inhibiting SER progression by a change of SER of 0.06 D by RLRL treatment in a 6-mouth trial reported by Dong et al 218, a change by 0.70 D in a 9-month trial from another report by Zhou et al 219 and a change by -0.59 D in a 12-month trial reported by Jiang et al 20. In addition, time spent outdoors at school (40 more minutes a day for 3 years) can bring a change of SER 0.17 D presented by He et al 91, DIMS may bring a change of SER (-0.44 D) in a 2-year study reported by Lam et al 24, high add power contact lenses may bring a change of SER (0.46 D) in a 3-year study presented by Walline et al 228, spectacle lenses with aspherical lenselets may bring a change of SER (0.53 D) in a 1-year study reported by Bao et al 229, 0.01% atropine treatment for 1 year may bring a change of SER (0.26 D) presented by Wei et al 230 and 0.025% atropine for 2-year treatment can bring a change of SER change (-0.85 D) in LAMP (phase II) investigation reported by Yam et al 213. Furthermore, the efficacy for inhibiting AL elongation may bring a change of axial growth by 0.11 mm in a 6-mouth RLRL trial by Dong et al 218, by 0.20 mm in a 9-mouth trial from another RLRL therapy by Zhou et al 219. RLRL may also reduce AL elongation by 0.26 mm in a 12-mouth RLRL trial by Jiang et al 20. Interestingly, time spent outdoors at school (40 more minutes a day for 3 years) may bring a change of axial growth by -0.03 mm presented by He et al 91, 0.01% atropine treatment for 1 year may bring axial growth change by 0.09 mm 230, and 0.025% atropine treatment for 1 year may lead to AL change by 0.29 mm in LAMP study by Yam et al 22 and 0.025% atropine treatment for 2-year may bring axial growth change by 0.50 mm (average 0.25 mm per year) in LAMP (phase II) study by Yam et al 213 and DIMS may bring a change of axial growth by 0.34 mm in a 2-year study (average 0.17 mm per year) by Lam et al 24 and high add power contact lenses may bring a change of axial growth by 0.42 mm in a 3-year study (average 0.14 mm per year) by Walline 228 and spectacle lenses with aspherical lenselets may bring a change of AL by 0.23 D in a 1-year study by Bao et al 229. All evidence is clear that RLRL treatment may effectively retard myopia progression reported by Jiang et al, Zhang et al, Dong et al, Zhou et al and Xiong et al 20, 30, 218-220, without severe adverse events observed by Jiang et al 20. As expected, compared to 0.01% atropine eye drops for myopia control, RLRL is more effective to retard AL elongation and myopia progression for 12 months observed by Chen et al 231. Xiong et al have evaluated the long-term efficacy as well as safety of RLRL therapy over myopia control for 2 years and potential rebound effect after withdrawal of treatment and discovered that RLRL therapy has encouraging efficacy and safety for retardation of myopia progression without serious side effects over 2 years reported by Xiong et al 220. Therefore, RLRL is an effective method to prevent and control myopia, probably with a slight myopic rebound after its withdrawal presented by Chen et al 232. Dong et al have noted that the efficacy and safety of RLRL therapy for retarding myopia progression requires 100% original power to significantly reduce myopia progression over 6 months, compared to that from only 10% original power. Jiang et al have reported that RLRL therapy is a promising treatment for myopia control in children 20. Further validation in long term randomized controlled trials are required for validation of the short-term trial results shown by Zhou et al 219. The results for recent clinical trials of RLRL therapy to myopia are listed in Table 1.

## Controversies

Effects of various lights on myopia may be quite different. For instance, red-light, a long wavelength light should lead to hyperopia defocus 50, 159, 236 and induce myopia 30. However, it is not always the case. The effect of red-light on progression of myopia is quite different. For instance, some researchers have reported that the predominant effect of red-light is to induce hyperopia but not myopia 51, 52. Little or no difference has been found in refractive error in a rhesus monkey model with or without quasi monochromatic red-light 75 while rhesus monkeys with long red wavelength filters show significant hyperopia 51. These results suggest that chromatic signaling is not required in certain cases 75. Reduction of the luminance contrast may lead to chromatic contrast mainly in emmetropization 61, 237, causing imbalance in strengths of short and long wavelength signals 51. Other reports have suggested that spectral wavelength and specific wavelength ranges may significantly influence myopia progression. For example, 630 nm light and 624 nm light may significantly affect myopia progression in rhesus monkey and tree shrew models 212, 238. RLRL at 650 nm and 1600 lx may significantly inhibit progression of myopia in school children 20. Tian et al have also concluded that 650 nm RLRL therapy may significantly retard myopia progression in children of 6-12 years old, without serious side effects in a 6-month trial 233. Yang et al have demonstrated that 650 nm RLRL therapy for 3 min has had no effect on the choroid but had effect with a transient increase of retinal fovea perfusion density 234. Low intensity and long wavelength red-light (635 nm) may also inhibit myopia progression in school children in Eastern China 219.

## Prospectives

Myopia has been increasing steadily, especially in East Asia. Red-light may improve choroidal blood perfusion and may be a critical tool for control of myopia via cytochrome and nitric oxide signaling. In all, application of red-light may retard myopia effectively, possessing high potential for prevention of myopia onset and control of myopia progression. However, the safety and the implementation of RLRL therapy are two biggest issues for extensive investigations. In addition, whether such a therapy is effective in high myopia requires further scientific studies. Furthermore, whether RLRL therapy can prevent onset of myopia remains unknown. Finally, whether RLRL therapy can be used to replace atropine therapy for myopia control needs comparative studies.

## Figures and Tables

**Table 1:** Summary of Recent Clinical Trials for RLRL Therapy to Myopia.

Authors	Year	Type of Studies	Study Duration (Months)	Sample Size	Efficacy and Study Results	Limitations
Zhou et al 219	2022	Retrospective case series	9	105	At 9 months, the mean SER in RLRL group was -2.87 ± 1.89 D, significantly greater than that of the control (-3.57 ± 1.49 D). AL changes were -0.06 ± 0.19 mm and 0.26 ±0.15 mm in RLRL group and the control group. The subfoveal choroidal thickness changed by 45.32 ± 30.88 µm in RLRL group at the 9-month examination. Specifically, a substantial hyperopic shift (0.31 ± 0.24 D and 0.20 ± 0.14 D) was found in 8-14 years old, compared with 4-7 years old children. The decrease in AL in subjects with baseline AL >24 mm was -0.08 ± 0.19 mm, significantly greater than those with a baseline AL ≤24 mm (-0.04 ± 0.18 mm). The authors have concluded that repetitive exposure to RLRL therapy was associated with slower myopia progression and reduced axial growth after short durations of treatment.	Firstly, selection bias might be present because not all participants adhered strictly to treatment. Secondly, the sample size was small and the duration was short (9 months). The sample size of the control group and experimental groups was not the same or similar. Thirdly, the wavelength and power intensity used in this study was different from others, making it difficult to compare the results with previous studies.
Jiang et al 20	2022	Randomized controlled trial	12	264	Adjusted 12-month axial elongation and SER progression were 0.13 mm (0.09-0.17mm) and -0.20 D (-0.29 to -0.11D) for RLRL treatment and 0.38 mm (0.34-0.42 mm) and -0.79 D (-0.88 to -0.69 D) for SVS treatment. The differences in axial elongation and SER progression were 0.26 mm (0.20-0.31 mm) and -0.59D (-0.72 to -0.46 D) between the RLRL and SVS groups. No severe adverse events (sudden vision loss ≥2 lines or scotoma), functional visual loss indicated by BCVA, or structural damage seen on OCT scans were observed. The authors have concluded that RLRL therapy is a promising alternative treatment for myopia control in children with good user acceptability and no documented functional or structural damage.	Firstly, because of pragmatic feasibility, the authors did not implement masking. Secondly, the level of compliance was not assigned randomly. Thirdly, because of coronavirus disease outbreak in 2019, approximately 50% of children were lost to follow-up at 6 months. Fourthly, the observed treatment efficacy in controlling myopic progression was generalizable only to the device used in the present study, not approved by studies using other wavelengths, power intensities, exposure durations, or frequencies of treatments. Fifthly, the duration of the trial was 1 year, which was not long enough.
Chen et al 231	2022	Randomized controlled trial	12	62	The mean 1- year change in AL was 0.08 mm in RLRL group and 0.33 mm in low dose atropine (LDA) group, with a mean difference (MD) of -0.24mm. The 1-year change in SER was-0.03 D in the RLRL group and -0.60 D in LDA group. The progression of AL *<* 0.1 mm was 53.2% and 9.7% in the RLRL and LDA groups, respectively. For AL ≥ 0.36 mm, progression was 9.7% and in the RLRL and LDA groups, respectively. The authors have concluded that RLRL is more effective for controlling AL and myopia progression over 12 months of use compared with 0.01% atropine eye drops.	Firstly, the authors might not avoid the deliberate exclusion of false reports from parents. Secondly, this study was a localized but not a multicenter study, and the geographic representation was not great. Thirdly, only 0.01% atropine was used to compare the results from RLRL study, which is not the balanced dose for control of myopia by atropine. Fourthly, due to covid-19, online learning may bias the results in the study. Fifthly, the duration was 12 months, not long enough for a definite conclusion from this study.
Xiong et al 220	2022	Post-trial follow up	24	199	In the second year, the mean changes in AL were 0.28 ± 0.14 mm, 0.05 ± 0.24 mm, 0.42 ± 0.20 mm and 0.12 ± 0.16 mm in SVS-SVS, SVS-RLRL, RLRL-SVS and RLRL-RLRL group, respectively (p < 0.001). The respective mean SER changes were -0.54 ± 0.39D, -0.09 ± 0.55D, -0.91 ± 0.48D, and -0.20 ± 0.56D respectively. Over the 2-year period, axial elongation and SER progression were smallest in RLRL-RLRL group (AL: 0.16 ± 0.37 mm; SER: -0.31 ± 0.79D), followed by SVS-RLRL (AL: 0.44 ± 0.37 mm; SER: -0.96 ± 0.70D), RLRL-SVS (AL: 0.50 ± 0.28 mm; SER: -1.07 ± 0.69D) and SVS-SVS group (AL: 0.64 ± 0.29 mm; SER: -1.24 ± 0.63D). No self-reported adverse events, functional or structural damage was noted. The authors have concluded that continued RLRL therapy sustained promising efficacy and safety in slowing myopia progression over 2 years. A modest rebound effect was noted after treatment cessation.	Firstly, the study was a post-trial follow-up research, and the participants were not randomly assigned to control and experimental groups. Secondly, the continuation or cessation of the treatment was not pre-determined, which might cause the bias in the results. Thirdly, the number of participants in the control and RLRL therapy groups was not balanced. Fourthly, the study was based on Chinese children, therefore, the generalizability of this study to other ethnicities needs further exploration.
Dong et al 218	2022	Randomized controlled trial	6	111	The mean SER change over 6 months was 0.06 ± 0.30 D in the RLRL group and -0.11 ± 0.33 D in the sham device control group, with respective mean increases in AL of 0.02 ±0.11mm and 0.13 ± 0.10 mm. In the multivariate GEE models, children in the RLRL group showed less myopia progression and axial elongation than those in the sham device control group (SER: coefficient, 0.167 D; 0.050-0.283D; AL: coefficient, -0.101 mm; -0.139 to -0.062 mm). No treatment-related adverse events were reported. The authors have concluded that in myopic children, RLRL therapy with 100% power significantly reduced myopia progression over 6 months compared with those treated with a sham device of 10% original power. The RLRL treatment was well tolerated without treatment-related adverse effects.	Firstly, the duration of this trial was 6 months, not sufficient for the observations of the full myopia control effects. Secondly, the rebound effect of RLRL treatment was not present. Thirdly, online screen-based learning might have affected the results of myopia due to covid-19. Fourthly, in the control group, the authors used 10% of RLRL energy, which might not be appropriate since 10% of RLRL energy might also have certain effects. Fifthly, masking of participants was not strict.
Liu et al 211	2022	Randomized controlled trial	1	98	A linear mixed-effects model showed that the AL of the subjects in RLRL decreased from 24.63 ±1.04 mm to 24.57 ± 1.04 mm, and the SChT thickened by 18.34 µm. CVI had a slight but significant increase in the 0-6 zone. However, all the anterior segment parameters did not change after RLRL treatment. The authors have concluded that choroid's thickening is insufficient to explain the AL shortening. The unchanged anterior segment and improved choroid blood flow suggest that AL shortening is related to changes in the posterior segment.	Firstly, the follow-up duration for this study is short. Secondly, the authors did not know how AL would rebound after discontinuation of their RLRL treatments in myopia. Thirdly, it is unclear how the short-term axial shortening links to long-term myopia control. Fourthly, it is unclear whether short-term axial shortening occurs in emmetropic subjects or only in myopic adults.
Tian et al 233	2022	Randomized controlled trial	6	224	The median 6-month changes in AL of the LLRL and control groups were - 0.06 mm and 0.14 mm, respectively. The difference between groups was significant. The median 6-month changes in SER were 0.125 D and -0.25 D for the LLRL and control groups, respectively. The difference between groups was significant. Compared with the control, the proportion of children with hyperopic shift in the LLRL group was higher (51.65% vs. 3.41%), and the proportion of children with shortened AL in the LLRL group was higher (63.74% vs. 2.27%). No adverse event was observed. The authors have concluded that 650 nm LLRL significantly slowed down the myopia progression in children aged 6-12 years, and there was no observable side effect in the short term.	Firstly, the duration of this study was 1 year, and the cessation time was 3 months, not long enough for the controlling effect on myopia. Secondly, the sample size is not good enough to draw a scientific conclusion. Thirdly, not all examination results were objective, such as the examination for accommodation. Fourthly, LRL recorded was subjective.
Yang et al 234	2022	Prospective study	1	25	The RFPD in LLRLT eyes significantly increased 5 min after LLRLT, and the increment was 1.70 ± 0.83%. The RFPD significantly decreased from 5 min to 1 h after LLRLT with a mean of -2.62 ± 0.86% decrement. The RFPD levels returned to baseline at 1 h after LLRLT. However, compared with insignificant RFPD changes in non-LLRLT eyes, there was no significant difference in RFPD changes at any sampling point. No significant changes in RFT, CFBF, and CFT were found in LLRLT eyes at each sampling point. Although 3 min of LLRLT has no effect on the choroid, it may cause a short-term transient increase in RFPD. The authors have concluded that their study provides theoretical support for the role of LLRLT in myopia control.	Firstly, the follow-up duration in this study was short, therefore, the long-term efficacy and safety of RLRL for myopia require further investigation. Secondly, the rebound effect was not determined.
Chen et al 232	2022	Randomized controlled trial	12	86	AL elongation and myopic progression were 0.01 mm (- 0.05 to 0.07 mm) and 0.05 D (- 0.08 to 0.19 D) in the LRL group, which were less than 0.39 mm (0.33 to 0.45 mm) and - 0.64 D (- 0.78 to - 0.51 D) in the SFS group. The change of SFCT in the LRL group was greater than that in the SFS group. Accommodative response and positive relative accommodation in the LRL group were more negative than those in the SFS group. Forty-two subjects completed the observation of LRL cessation, AL and SER increased by 0.16 mm (0.11 to 0.22 mm) and - 0.20 D (95%- 0.26 to - 0.14 D) during the cessation, and SFCT returned to baseline. The authors have concluded that LRL is an effective measure for preventing and controlling myopia, and it may also have the ability to improve the accommodative function. There may be a slight myopic rebound after its cessation.	Firstly, only 25 cases were included in this study, which might cause the bias in their conclusions. Secondly, the data of RLRL therapy was from only one device, which needs confirmation from data obtained from other machines because different devices for RLRL therapy might have other power intensities and wavelengths. Thirdly, the appropriate exposure durations were not determined.
Wang et al 235	2023	Retrospective study	12	434	The mean age of participants was 9.7 (2.6) years with SER of -3.74 (2.60) diopters. There were 115 (26.50%), 76 (17.51%), and 20 (4.61%) children with AL shortening based on cutoffs of 0.05 mm/year, 0.10 mm/year, and 0.20 mm/year, respectively. In the multivariable model, AL shortening was significantly associated with older baseline age, female gender, and longer baseline AL or greater SER. Among AL shortened eyes, the mean AL difference was -0.142 (0.094) mm/year. Greater AL shortening was observed among children who were younger and had longer baseline AL. The authors have concluded that more than a quarter of children have had AL shortening [0.05 mm following RLRL therapy, and the overall mean AL change was -0.142 mm/year.	Firstly, the children were Chinese ethnicity, which might not be representative in other geographic regions of the world. Secondly, this study duration is not long enough for follow-up study of RLRL therapy on the control of myopia. Secondly, AL measurements might be affected by the changes in choroidal thickness. Thirdly, no data on outdoor activities on myopia was available in the retrospective study, which could cause significant bias. Fourthly, the study duration is 1 year for follow-up information, not sufficient for an estimate of myopia reduction that should take a few years to complete.
